# Luck, lottery, or legacy? The problem of confounding. A reply to Harden

**DOI:** 10.1111/evo.14588

**Published:** 2022-09-06

**Authors:** Graham Coop, Molly Przeworski

**Affiliations:** ^1^ Center for Population Biology and Department of Evolution and Ecology University of California Davis CA USA; ^2^ Department of Biological Sciences and Department of Systems Biology Columbia University New York NY USA

## Abstract

A reply to Harden's response to Coop and Przeworski (2022).

In Coop and Przeworski (2022), we argue that Harden (2021) misinterprets findings from genome‐wide association studies (GWAS) of educational attainment and overstates their relevance for our understanding of social inequalities. We focus on three key steps of the book's central argument: (1) the reliance on the metaphor of polygenic scores as drawn from a “genetic lottery” analogous to a randomized controlled trial; (2) the attribution of all GWAS signals to the brain of the focal individual; and (3) the treatment of GWAS findings as reflecting genetic causes of inequality within an ancestry group, but as uninterpretable between ancestry groups. Each of these three steps is invalid, for largely the same reason: not following through on what it means for the genetic effects identified in GWAS to be confounded by genomic background and by environmental effects correlated with that background. Thus, the *“meta‐message”* of our review is not, as Harden (2022) writes in her reply, that *“It's complicated”* but rather that “It's confounded.”

This confounding may not preclude uses of polygenic scores for educational attainment as control variables in the social sciences. But it does mean that these scores cannot be interpreted as identifying genetic causes of inequality. There is a long history of using confounded biological and genetic measures to explain poverty and inequality. Although Harden stakes out a different political position, her book nevertheless participates in that same tradition by downplaying the limitations of GWAS.

Here, we clarify some erroneous statements about our review and the GWAS literature in her reply. We also note some distortions, including in the quotations. As an example, Harden cites us as saying *“we …fully grant the book's starting point …GWAS [genome‐wide association studies] undoubtedly capture some causal genetic effects.”* The quotation provided combines two sentences that are five pages apart in our review; what we write at the outset is, in fact: *“that educational attainment is heritable was documented before GWAS and is in some sense trivial …We thus fully grant the book's starting point.”*

## THE ELISION OF DIFFERENT TYPES OF GWAS

GWAS associate trait variation with genetic loci. In controlled or randomized environments, those associations can help to identify genetic causes. In human GWAS, the hope is to disentangle genetic and environmental effects through the use of statistical controls. These controls are not perfect, however, and for some traits, notably behavioral ones, substantial confounding of genetic effects remains (Kong et al., 2018; Mostafavi et al., 2020; Selzam et al., 2019). That problem can be mitigated in family‐based designs: the randomization of genotypes transmitted from parents to offspring allows direct genetic effects‐the effects of alleles carried by a person on that person‐to be teased apart. But a standard GWAS does not have that property. Instead, associations identified in a standard GWAS absorb indirect genetic effects and numerous confounders. As summarized in a recent review article: *“There are at least three sources of confounding in GWAS: (i) environmental confounding, where allele frequencies and environmental effects vary in a correlated way across different geographic regions…; (ii) genetic confounding…or (iii) assortative‐mating confounding…These forms of confounding are conceptually different, but in practice they are often intertwined. ”* (Young et al., 2019). To date, almost all GWAS have been of the standard design. Therefore, despite Harden's emphasis on causal identification and genetic lotteries—per the title—almost all of the genetic evidence marshaled in the book is potentially confounded, often to an unknown degree (Fletcher, 2022). Harden notes some of these limitations at various points, but inconsistently, and without pursuing their implications for her central claims.

In her reply, Harden acknowledges limitations in drawing inferences about differences between families from sib‐GWAS, but incorrectly argues that these do not extend to trio studies or other family‐GWAS designs. In reality, the same limitations apply. As noted in Young et al. (2019), despite some technical differences, all nuclear family‐GWAS designs similarly use *“parental genotypes as controls to separate direct from indirect genetic effects and other confounding effects.”* In some contexts, those direct genetic effects can be interpreted as causes. When it comes to interpreting trait differences between people from different families, however, or to predicting traits, all the effects come into play, not just direct effects (Becker et al., 2021; Okbay et al., 2022; Young et al., 2022).

Harden also describes a related method inaccurately in her reply, writing that *“Relatedness disequilibrium regression (RDR)…was described by Dr. Przeworski in one of her previous co‐authored papers as giving ‘unbiased estimates of direct genetic effects’ (Young et al.*, 2019).” The Young et al. quote is actually not a definition of RDR, about which the paper contains only one description, in the legend of Figure 4: as providing *“an estimate of the SNP heritability using a within family method”* (Young et al., 2019). As for Harden's claim that *“Results from RDR suggest that, yes, genetic influences account for variation in educational attainment, and not just within‐family variation,”* we are unsure what she has in mind, other than a loose restatement of RDR being a method to estimate heritability.

For educational attainment specifically, confounders are a major concern. Indeed, the RDR estimate of heritability, which is designed to be less inflated by environmental confounders, is substantially lower than other estimates (Young et al., 2018; Figure 4 in Young et al., 2019). In her book, Harden provides numbers for various correlates of educational attainment, but never reports how much of the GWAS signal and the resulting polygenic prediction for educational attainment have been attributed to direct genetic effects versus other sources, such as indirect effects and population‐structure confounding. In her reply, she refers only to one meta‐analysis (Wang et al., 2021), on the basis of which she states that *“there are ‘indirect’ genetic effects on educational outcomes* (β*= .08, 95% CI = .07 to .09), but these indirect genetic effects are smaller than the ‘direct’ genetic effects* (β*= .17, 95% CI = .13 to .20).”* These numbers are meant to represent the effects (regression coefficients) of direct versus indirect effects on educational attainment. But the estimate reported for “indirect effects” is actually for only one of two parents (Wang et al., 2021); moreover, it includes potential effects of confounding which, alongside other statistical issues, complicates the comparison to direct genetic effects (Fletcher et al., 2021; Mostafavi et al., 2020; Trejo & Domingue, 2018; Wang et al., 2021; Young et al., 2022).

Multiple papers that go unmentioned explicitly decompose the variance in educational attainment explained by polygenic scores. These studies find that in European‐ancestry individuals, as little as a quarter to a third of the variance explained by the polygenic score is attributable to direct genetic effects, with the other two‐thirds to three‐quarters ascribed to a complex mixture of indirect genetic effects and population‐structure confounders (Howe et al., 2022; Mostafavi et al., 2020; Okbay et al., 2022; Young et al., 2022). Thus, the latest GWAS for educational attainment reports that, although  12‐16% of the variance in educational attainment among people of European ancestry is predicted by polygenic scores, less than 5% of the variance is due to direct genetic effects (Okbay et al., 2022). A recent paper, which Harden co‐authored, attempts to tease apart parental indirect effects and confounders by analyzing siblings of the parents as well as nuclear families; it concludes that much of the non‐direct genetic effects may be due to *“dynastic stratification in environments relevant to success in school”* (Nivard et al., 2022), a form of confounding.

## THE INTERPRETATION OF GWAS HITS FOR EDUCATIONAL ATTAINMENT AS “BUILT IN” DIFFERENCES AMONG INDIVIDUALS

In her book, Harden misinterprets the GWAS enrichment analysis to imply that all GWAS associations can be ascribed to genes active in the brain, writing, e.g., that *“Whatever genes are doing to make it more or less likely for some people to succeed in education, they are doing it in people's brains, not their hair or livers or skin or bones.”* [p. 137]. In fact, the gene enrichment analysis for the GWAS simply shows that the associations are found more often than expected by chance near genes also expressed in the brain; it does not rule out causal effects mediated by other tissues.

Elsewhere, she writes that: *“Thousands upon thousands of genetic variants matter for educational attainment …These genes exert their effects via largely unknown cellular processes that are happening in neurons and other brain cells. These cellular effects are already happening during prenatal development, and their effects on the individual organism are already evident in childhood …”* [p. 148]. In so doing, she repeatedly uses phrasing that ascribes the entire predictive power of a standard‐GWAS polygenic score to the activity of genes in the brain of the focal individual, which again misinterprets gene enrichment analyses, and ignores the fact that much of the GWAS signal stems from indirect effects and confounding.

Harden does not address these points in her reply, and instead argues that *“it is doubtful that any credible account of the causal paths from genetic differences between people to educational inequality will somehow circumvent psychological differences between people, or that these psychological differences will somehow not involve the brain.”* Psychological traits undoubtedly play a role in educational attainment; that was never in dispute. The issue is rather that enrichment analyses and related studies leave open other causal genetic paths that are not about psychology: for instance, children at risk for chronic childhood diseases may tend to fall behind in their schooling. Moreover, Harden's argument once again ignores the consequences of confounding: namely that genetic differences among individuals identified by GWAS cannot be assumed to be causally related to educational attainment. As just one example: to the extent that the GWAS is confounded by dynastic stratification in environments, psychological differences may not explain why children find themselves trapped in under‐performing schools.

## THE RELIANCE ON TYPOLOGICAL NOTIONS OF POPULATIONS

We point out in our review that Harden cannot have it both ways: she cannot simultaneously ignore the limitations of GWAS when it is conducted in individuals of European ancestry and yet invoke them to forestall comparisons of GWAS findings between ancestry groups. In her reply, Harden acknowledges the lack of any clear boundary between ancestry groups and the difficulty of controlling for confounders, but argues that these are unavoidable features of the social sciences, adding *“The study of environmental influences is every bit as difficult [as that of genetic influences]. For many environments of interest, experimentation is impossible.”* We agree: the lack of control or randomization of the environment only makes it that much more difficult to interpret polygenic scores for educational attainment from a standard GWAS. As a consequence, the big jump is not from one “ancestral population” to another, as described in her book, but from within‐ to between‐family comparisons, where one loses the ability to tease apart genetic effects from environmental ones.

Harden writes that *“Because of the difficulty of that ‘big jump’, Coop and Przeworski conclude that ‘current PGS [polygenic scores] for educational attainment are neither interpretable nor meaningful.”'* In point of fact, we wrote that current educational attainment polygenic scores are *“neither interpretable nor particularly meaningful”* because, even for direct effects, there is little understanding of the developmental or physiological mechanisms through which they act (in addition to the usual problem of causal loci rarely being distinguished from the variants that tag them). Nor has the field yet figured out where up to three‐quarters of the standard GWAS signal is coming from.

In her reply, Harden does not mention that existing polygenic scores for educational attainment were developed based on GWAS‐enrollees of European ancestry and have substantially reduced prediction accuracy in individuals of less similar genetic ancestry (Martin et al., 2019; Mills & Rahal, 2020; Okbay et al., 2022; Privé et al., 2022). In her book, she does, and moving from ancestry groups to racial labels, she describes a near future in which we will have a polygenic score *“that is as strongly related, statistically, to academic achievement in Black students as it is in White students.”* [p. 191] (This conflation of ancestry and race is particularly consequential when considering Black Americans, who vary greatly in their proportions of recent African and European ancestries, in ways that are correlated with both geography and economic opportunity (e.g., Baharian et al., 2016; Micheletti et al., 2020).) Harden warns the reader, however, that such comparisons between Black and White Americans would be *“scientifically and ethically wrong”* [p. 192]. To explain why comparisons of genetic effects between groups are invalid, and will remain so even when polygenic scores have been constructed for both, she refers to statistical issues that arise from confounders and the lack of environmental homogeneity. But as we outline in our review, similar problems, differing in degree rather than in kind, apply to the move from within‐family studies to standard GWAS.

Harden interprets our charge that she is trying to have it both ways as somehow related to polygenic scores being *“simultaneously meaningful…and limited, by virtue of their contextual dependence and imperfect portability”* , adding *“As the author Maggie Nelson wrote: ‘There is a lot to be learned from having it both ways' ”*. In reality, we were making the point that she cannot overlook the effects of confounding when interpreting polygenic scores within European‐ancestry GWAS individuals—let alone within the racial grouping of “Black Americans”—only to resurrect them when seeking protection against the implications of her claims for between ancestry‐group comparisons. The truth is that polygenic scores for educational attainment are also confounded within ancestry groupings, in ways that greatly complicate interpretation.

## IMPLICATIONS

In concluding our review, we caution that the interpretability of polygenic scores for educational attainment *“matters greatly when they are used to elucidate, let alone redress, social inequalities.”* Harden recasts our statement in terms of ethical commitments, writing: *“I am wary of the review's conclusion that ‘it matters greatly’ whether the causal paths connecting genetics to educational attainment are … physical phenotypes such as skin or hair color that society discriminates against, rather than mediated through the development of neurocognitive phenotypes …Are people's claims on society for their inclusion and participation, regardless of difference, any less valid if the difference is psychological versus physiological? I think not.”* Of course not. Where the interpretability of polygenic scores matters greatly is if, as Harden advocates in her book, they are to be a basis for putting ethical commitments into practice.

We also convey our *“doubt[s] that overstating our understanding of the genetics of behaviors is going to increase empathy.”* In her reply, Harden ignores the word “overstate”, arguing that *“research has shown that ‘understanding of the genetics of behaviors,’ ranging from sexual orientation to body size to serious mental illness, can indeed increase people's empathic attitudes, reducing ascriptions of blame and increasing support for civil rights.”* She contends that what would really convince Americans and in particular *“American conservatives”* to support greater equality is evidence from behavioral genetics. The two references provided by Harden in support of her claim are more circumspect, concluding that *“biogenetic explanations for mental disorders confer mixed blessings for stigma”* (Kvaale et al., 2013) and that *“persuasion tended to be conditioned on citizens’ political values”* (Garretson & Suhay, 2016).

As scientists, our responsibility is to be deliberate about the questions we pose and transparent about the strength of our findings. That includes asking ourselves whether, as geneticists, we may end up overstating the utility of genetics in addressing social problems, recasting structural and historical inequities as embodied or psychological characteristics of individuals. In that regard, when the goal is addressing inequality, it seems worthwhile to take a broader perspective than that of the US. Many countries redistribute income and develop social programs to a greater extent than does the US, without invoking genetics, let alone GWAS.

1

Associate Editor: Dr. J. Mallet

Handling Editor: Dr. T. Chapman
